# Plasticity in Cervical Motor Circuits following Spinal Cord Injury and Rehabilitation

**DOI:** 10.3390/biology10100976

**Published:** 2021-09-28

**Authors:** John R. Walker, Megan Ryan Detloff

**Affiliations:** Marion Murray Spinal Cord Research Center, Department of Neurobiology & Anatomy, College of Medicine, Drexel University, Philadelphia, PA 19129, USA; jw3646@drexel.edu

**Keywords:** primary afferents, nociceptor, reach-to-grasp, forelimb function, upper extremity function

## Abstract

**Simple Summary:**

Spinal cord injury results in a decreased quality of life and impacts hundreds of thousands of people in the US alone. This review discusses the underlying cellular mechanisms of injury and the concurrent therapeutic hurdles that impede recovery. It then describes the phenomena of neural plasticity—the nervous system’s ability to change. The primary focus of the review is on the impact of cervical spinal cord injury on control of the upper limbs. The neural plasticity that occurs without intervention is discussed, which shows new connections growing around the injury site and the involvement of compensatory movements. Rehabilitation-driven neural plasticity is shown to have the ability to guide connections to create more normal functions. Various novel stimulation and recording technologies are outlined for their role in further improving rehabilitative outcomes and gains in independence. Finally, the importance of sensory input, an often-overlooked aspect of motor control, is shown in driving neural plasticity. Overall, this review seeks to delineate the historical and contemporary research into neural plasticity following injury and rehabilitation to guide future studies.

**Abstract:**

Neuroplasticity is a robust mechanism by which the central nervous system attempts to adapt to a structural or chemical disruption of functional connections between neurons. Mechanical damage from spinal cord injury potentiates via neuroinflammation and can cause aberrant changes in neural circuitry known as maladaptive plasticity. Together, these alterations greatly diminish function and quality of life. This review discusses contemporary efforts to harness neuroplasticity through rehabilitation and neuromodulation to restore function with a focus on motor recovery following cervical spinal cord injury. Background information on the general mechanisms of plasticity and long-term potentiation of the nervous system, most well studied in the learning and memory fields, will be reviewed. Spontaneous plasticity of the nervous system, both maladaptive and during natural recovery following spinal cord injury is outlined to provide a baseline from which rehabilitation builds. Previous research has focused on the impact of descending motor commands in driving spinal plasticity. However, this review focuses on the influence of physical therapy and primary afferent input and interneuron modulation in driving plasticity within the spinal cord. Finally, future directions into previously untargeted primary afferent populations are presented.

## 1. Introduction

The negative consequences of spinal cord injury (SCI) arise from far more than the loss of directly damaged grey matter and neural pathways. Unfortunately, these dead and dying neurons release death signals which exacerbate the injury. In response to damaged and dying tissue, the innate and adaptive immune response will become activated as described in detail by Donnelly and Popovich [1]. Monocyte-derived macrophages and activated microglia clear debris from the initial primary insult. However, these immune cells remain long after debris is cleared and provide a continual bombardment of inflammatory cues that initiate secondary injury in areas rostral and caudal to the injury epicenter [2,3,4,5,6]. In an effort to mitigate secondary injury, reactive astrocytes physically limit the spread of inflammation, compensate for a leaky blood brain barrier, and reduce lesion expansion by forming a glial scar [7,8,9]. However, this physical barrier may also prevent axonal regeneration through the lesion. Reactive astrocytes upregulate signal transducer and activator of transcription 3 (STAT3) and release chondroitin sulfate proteoglycans (CSPGs) and bone morphogenic protein (BMP), inhibiting the growth of neurons and oligodendrocyte maturation and subsequent remyelination efforts [9,10,11,12,13,14,15,16]. Evidence does support astrocytic release of growth promoting factors, such as laminin [7,17]; however, the cumulative effect is detrimental to recovery. Other processes also contribute to the inability of damaged spinal cord axons to regenerate after injury. Wallerian degeneration of the distal axons and myelin results in debris releasing Nogo, OMGp, and MAG, which have all been shown to inhibit regeneration and sprouting [18]. Collectively, these impediments limit the efficacy of spontaneous recovery.

Thus, for the vast majority of individuals, SCI results in permanently lost ascending and descending neuronal connections that are important for normal behavior and function, even with existing treatments and rehabilitation paradigms [19]. Injury-induced damage and failed regeneration necessitates that the remaining central nervous system (CNS) compensates for lost function. Depending on the type and location of injury, damaged and undamaged neurons will show sprouting, new synapse formation, and changes in electrophysiological properties. However, in the case of a complete SCI, in which there are no spared connections, the loss of long descending connections makes volitional control of movement impossible. Therapeutic interventions for individuals with complete SCI are limited to regenerative medicine or the utilization of compensatory devices [20,21]. Some spontaneous plasticity of the spared nervous system provides an avenue for recovery. Evidence for redundant and usually silent interneuron pathways have been shown in injury models, such as in the cross-phrenic phenomenon involved in the recovery of respiratory function [22,23,24]. Unfortunately, much of the injury-induced plasticity can be maladaptive, taking the form of aberrant sprouting and synaptogenesis as neurons try either to compensate for lost connections or to regenerate through the injury site as they respond to inflammation. Hyperexcitability and inefficiency result from these new connections, making restoration of normal function nearly impossible.

Plasticity is an incredible feature of the CNS, giving it the ability to learn and recover from insult. Without the guidance of rehabilitation, however, it yields limited functional improvements following SCI. Many rehabilitative interventions have been studied for their effects on recovery of function related to alterations in spinal cord anatomy and physiology [25]. Changes in the spinal cord do not just occur with action—i.e., motor output. Instead, rehabilitation- or activity-dependent plasticity of the spinal cord is thought to be afferent driven [26,27,28,29]. As the body moves or performs motor tasks, the spinal cord receives input about the quality of the movement from sensory neurons with receptors in the skin, muscles, and joints. Dorsal horn sensory neurons and interneurons receive this afferent input and refine connections, as well as output commands of the motor circuits. Furthermore, projection neurons from spinal cord motor centers provide feedback to supraspinal locations involved in modifying motor behavior (i.e., cerebellum, basal ganglia, motor cortex, etc.) [30,31,32,33]. Following an overview of the term “neuroplasticity,” this review will focus on the plasticity that occurs naturally following SCI, and how rehabilitative strategies enhance recovery of upper extremity or forelimb function through afferent driven and interneuron-mediated local plasticity in the spinal cord.

## 2. What Is Neuroplasticity?

Neuroplasticity is defined as the potential for functional and anatomical changes of the nervous system in response to stimuli during learning or in response to injury [34]. Our understanding of neuroplasticity has evolved over decades. Originally, experiments established the importance of circuits in behavior. Subsequent studies revealed how these synapses change with learning and the molecular mechanisms of these changes [35]. Classic experiments from the learning and memory fields described plasticity of the adult nervous system [36]. Receptor changes and the physical addition and/or subtraction of synapses modify synaptic efficiency, thereby driving neuroplasticity. These processes are responsible for learning, memory, and the fine-tuning of motor control. Evidence of neuroplasticity is found in both the spinal cord and the brain. The greatest occurrence of these changes happens during neurodevelopment. Until relatively recently, the dogma prevailed that synaptic connections within the adult nervous system were hardwired and fixed. Now, it is well established that the adult CNS can be modified, especially following injury.

One aspect of neuroplasticity is the strengthening and weakening of synapses in response to input. These synaptic changes are essential for learning, memory, and motor output under normal and pathological conditions. According to Hebbian theory, both pre- and post-synaptic neurons experience changes following repeated and persistent firing [37,38]. Presynaptic release probability, the number and properties of post-synaptic receptors, and the number of active synapses may all be altered [39,40,41].

**Figure 1:**
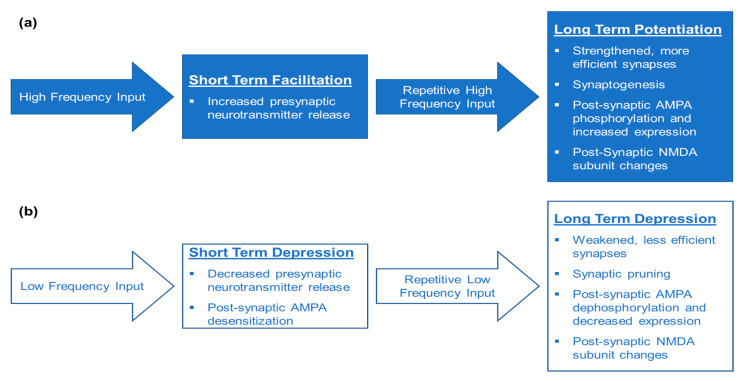
Key Characteristics of Neural Plasticity based on Hebbian learning. (**a**) Synaptic connections become stronger and more efficient following high frequency and repetitive input. This strengthening is known as either short-term facilitation or long-term potentiation. On a molecular level, single bouts of high frequency input result in increased neurotransmitter release, while repetitive bouts of high frequency input increases synaptogenesis, synaptic efficiency by modulating post-synaptic AMPA and NMDA receptor subunit expression and phosphorylation. These cellular and molecular changes are thought to underlie learning and memory, whether that be for episodic memory or refinement of motor control. (**b**) Synaptic connections can become weaker and less efficient after low frequency input. An episode of low frequency input results in short-term depression and is associated with decreased presynaptic neurotransmitter release, desensitization of AMPA receptors. Repetitive low frequency input results in long-term depression, which results in weakened, less efficient synapses, pruning of unused synapses, as well as dephosphorylation of AMPA receptors and changes in NMDA receptor subunit composition. Similarly to long-term potentiation, short- and long-term depression are also crucial aspects of learning and memory as unnecessary, redundant, and inefficient connections get pruned away to optimize the function.

In addition to alterations in synaptic strength, researchers have determined that plasticity within learning and memory circuits could occur for varying amounts of time depending upon the composition of pre- and post-synaptic sites. Transient plasticity is known as either short-term facilitation or depression [42,43] (Figure 1).

**Figure 2:**
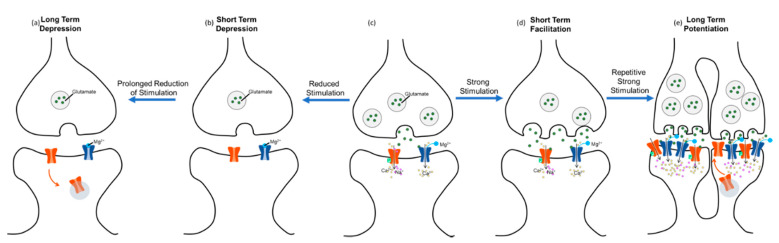
Synaptic Plasticity in Long-Term Potentiation (LTP) and Long-Term Depression (LTD). This figure depicts the cellular mechanisms of LTP and LTD starting from (**c**). During LTP (**c**–**e**), more frequent and greater stimulation releases glutamate form the presynaptic terminal, which binds to and opens AMPARs letting Na^+^ and Ca^2+^ enter the post-synaptic dendrite to depolarize the cell. Sufficient depolarization removes the Mg^2+^ from the NMDAR, which is also opened by glutamate, so that the NDMARs can detect the coincidence of activation between the two neurons and allow even greater influx of Ca^2+^ and Na^+^. This high concentration of intracellular Ca^2+^ leads to kinase activation, which in turn leads to the phosphorylation of AMPARs. Phosphorylated AMPARs stay open longer when glutamate binds, and more AMPARs are brought to the plasma membrane. Repetition of this process eventually leads to synaptogenesis. During LTD (**a**,**b**), less frequent and smaller release of glutamate binds to fewer receptors leading to a reduced influx of Ca and Na and prevents the removal of the Mg^2+^ plug. Lower Ca levels lead to phosphatase activation and the dephosphorylation of AMPARs, resulting in less time open and the internalization of Inactive AMPARS. LTD often leads to pruning of extraneous synapses.

Short-term facilitation results from changes in presynaptic neurotransmitter release [42,43,44] (Figure 2d), while short-term depression occurs when presynaptic neurons lack neurotransmitter vesicles to release into the synaptic cleft (Figure 2b). Postsynaptically, α-Amino-3-hydroxy-5-methyl-4-isoxazolepropionic acid (AMPA) receptors are desensitized [45]. Desensitization occurs when receptors have decreased responsiveness to stimuli. The processes of short-term facilitation and depression are primarily studied in the context of short-term memory and often precede the long-term changes.

Longer-lasting plasticity is known as long-term potentiation (LTP) or long-term depression (LTD). LTP occurs when synapses are strengthened and require less stimulation to propagate an action potential [46] (Figure 2e). Conversely, LTD occurs when synaptic strength is decreased, and more stimulation is required to propagate an action potential (Figure 2a). LTP and LTD are the mechanisms of long-term learning and memory. This learning is not limited to episodic memory; these processes also underlie changes in motor control circuitry in the motor cortex, cerebellum, and spinal cord [47,48,49]. Long-lasting plasticity within the spinal cord will be the focus of this review. The two main mechanisms of LTP in the spinal cord are receptor-mediated plasticity and synaptogenesis of either intact sprouting axons or the regeneration of damaged axons [50,51,52].

Changes in both AMPA and N-methyl-D-aspartate (NMDA) glutamate receptors on the post-synaptic neuron are a robust method for the nervous system to prioritize different connections.

In AMPA receptor-mediated plasticity, the neurotransmitter glutamate is released from the presynaptic terminal [39,53]. Glutamate then binds to NMDA and AMPA receptors on the post-synaptic neuron. AMPARs open first, allowing an influx of Na^+^. A sufficient influx of Na^+^ will depolarize the post-synaptic terminal and remove the Mg^2+^ block from NMDARs. This allows an influx of Ca^2+^ to the neuron, and the levels of Ca^2+^ influx are contingent on the degree and frequency of stimulation from presynaptic glutamate release. High stimulation and high Ca^2+^ influx will cause a phosphorylation cascade, resulting in phosphorylated AMPARs and more receptors being expressed on the plasma membrane. Phosphorylation makes AMPARs reach activation threshold with less stimulation and remain open longer [54]. These changes result in LTP.

Alternatively, lower levels of stimulation and Ca^2+^ influx will result in LTD. Low levels will activate phosphatases which dephosphorylate AMPARs. In this case, dephosphorylated AMPARs are less likely to open and will close sooner. Additionally, fewer receptors will be expressed on the membrane [54]. Although these processes are AMPA receptor-mediated, they are NMDA receptor-dependent because it serves as a coincidence detector for glutamate and depolarization. Alternatively, NMDA receptor-mediated plasticity involves a very similar process [44]. In this case, the NMDARs have subunit changes rather than phosphorylation events that determine their activity levels.

Synaptic sprouting and pruning are two major results of LTP and LTD. Synaptic sprouting, or synaptogenesis, often follows LTP [41,55]. Essentially, synapses experiencing sufficient LTP will split apart, creating two synapses. The post-synaptic density grows until it splits, and the presynaptic density will split to match. The split continues to perforate until the spine separates into multiple spines with multiple synapses. This physical change allows for a greater chance that excitatory post-synaptic potentials will summate into action potentials. Alternatively, LTD will result in pruning of synapses, and inactive synapses will eventually be eliminated [55]. Synaptogenesis and synaptic pruning both play a significant role in plasticity after injury.

## 3. Neural Plasticity Associated with Reaching and Grasping after SCI

**Figure 3:**
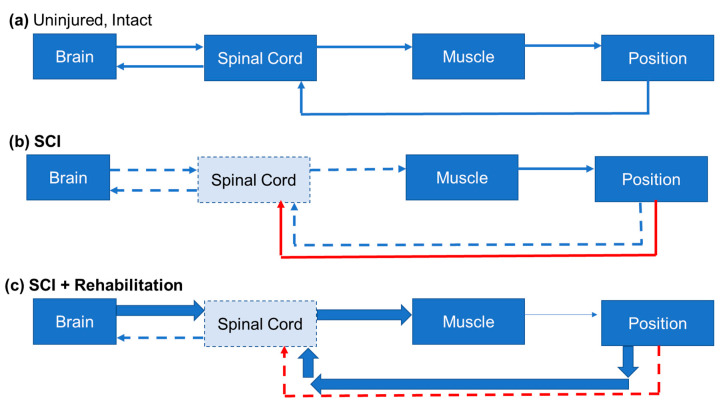
Motor and Sensory Input Through the Spinal Cord. This figure depicts a simplified model of motor output and sensory feedback used to guide movement in the intact CNS (**a**), after SCI (**b**), and during rehabilitation (**c**). (**a**) The final steps of motor planning involve a signal being sent from the motor cortex down to the motoneurons in the spinal cord. These motoneurons send signals to neuromuscular junction causing the appropriate muscle to contract and guide the limb to the final position. Feedback from the periphery regarding the final position gets sent back to the spinal cord. The spinal cord uses this information to refine the local connections and sends that information to brain regions involved in motor refinement, such as the cerebellum. (**b**) After injury, the corticospinal neurons lose some of their connections to the spinal cord (dashed lines), and the cord itself loses motoneurons. This results in a weaker signal to the muscle and mobility problems. Additionally, increased and maladaptive sensory input from primary sensory neurons, like the nociceptor, (red arrows) causes LTP related to central sensitization in chronic pain and noise to enter the system making refinement difficult. The sensory information has weakened connection to the brain. (**c**) Rehabilitation harnesses LTP (Figure 1) by forcing descending input from the brain, and sensory input from the periphery, to strengthen and refine connections withing the cord and motor cortex to regain mobility and decrease nociceptive input.

The reach-to-grasp movement is highly synchronous and composed of several observable components, including limb lifting, aiming, and advancing the limb, and followed by opening the digits, pronating the wrist, grasping the object, and supinating to orient the object for release into the mouth [56,57]. In humans, fine motor control of the digits is largely controlled by the descending lateral corticospinal tract (CST), which decussates and crosses midline at the pyramids in the brainstem, and then continues through the dorsolateral white matter of the spinal cord. These lateral CST fibers synapse in cervical motor pools in the spinal cord to control proximal and distal muscles of the limb and digits. The motor pools for the shoulder and arm are located at levels C4-6, and the motor pools of the forearm and digits are located in C7-T1 [58]. In addition to CST control in non-human primates, there is evidence of the involvement of descending rubrospinal and reticulospinal tract (RST) fibers in controlling which upper extremity muscles execute the reach and grasp of a target object [59,60,61,62]. Recently, direct excitatory projections from the deep cerebellar nuclei to the ipsilateral cervical spinal cord have been discovered to be involved in the control of the reach-to-grasp movement. Sathymurthy et al. [63] demonstrated direct cerebellospinal connections that were important for reaching and grasping. Mice with silenced ipsilateral cerebellospinal projection neurons took longer to touch the food pellet and failed to successfully grasp it. Rodent models have been extensively studied for reaching and grasping because of the many conserved movements and neuroanatomical substrates across species [64,65,66,67,68]. Forelimb behaviors are reliably measured in the laboratory using a combination of qualitative and quantitative assessments of reach-to-grasp pellet retrieval tasks [56,69], supination tasks [70], digit manipulation [71,72,73], and grooming behaviors [74]. After SCI, recovery or compensatory reaching and grasping is mediated by several spared systems that respond after injury. Sparing and sprouting of the CST and RST are two of the most well-characterized mechanisms involved in regaining reaching and grasping following SCI that are conserved across species [75,76,77,78,79]. In addition, plasticity of primary afferent fibers is also a key contributor to improved function post-injury. The following sections will focus on discoveries regarding both spontaneous and activity- or rehabilitation-driven plasticity in these pathways that mediate reaching and grasping movements (Figure 3). This review will conclude with our perspective on the specific role of sensory feedback in recovery and rehabilitation. 

### 3.1. Plasticity in Spared Descending Systems

The majority of descending input to upper extremity motoneurons in the cervical spinal cord come from the CST and extensive work has been done to map to its origin in the motor cortex [80,81]. In humans, the CST and the RST are responsible for control of the digits—the CST controls precision gripping, while the RST controls the power grasp [82]. Likewise, CST projections are conserved across species, including rats, non-human primates, and humans [83,84].

There are many examples of CST plasticity after SCI that include sprouting and the indirect control of motoneurons [85]. Weidner, et al. [86] showed that lesions of the corticospinal motor pathway in the high cervical spinal cord of rats led to significant sprouting of the contralateral ventral CST across midline into the ipsilesional medial motor column of Lamina IX and this anatomical plasticity was critical to post-injury gains in function. As in rats, non-human primates with unilateral cervical SCI demonstrated some improvement in reaching and grasping over time that corresponded with changes in the distribution of CST terminals in the spinal cord grey matter compared to intact macaques [87]. These CST axons rostral and caudal to the injury site terminate in Lamina VII, whereas the sprouting fibers synapse near motor pools in Lamina IX. Together, these data suggest that spontaneous plasticity of the spared components of the CST is a compensatory mechanism underlying forelimb motor recovery, as opposed to the restoration and/or regeneration of the damaged motor tract.

Spinal interneurons are key mediators of motor function. They integrate descending motor commands and sensory input from primary afferent fibers to modulate motor neuron activity and motor output [88,89]. There are diverse subpopulations of segmental spinal interneurons, many of which have been discovered in the context of understanding the neural control of locomotion and have been reviewed extensively elsewhere [90,91,92,93,94,95,96,97,98,99]. Long projecting propriospinal interneurons that connect cervical and lumbar enlargement are also important for interlimb coordination, especially during locomotion [100]. After SCI, these specialized spinal interneurons are a substrate for spinal plasticity and improved functional recovery. Their long axons allow descending motor commands to bypass lesion sites and reach distal motor pools [101,102,103,104].

Interneurons in the cervical cord are involved in many tasks, such as breathing, locomotion, and reach-to-grasp [97,105,106,107,108]. An example of propriospinal interneurons involved in the reach are the V2a interneurons, which relay information between motoneurons and the cerebellum to provide an ‘internal feedback loop’ [109]. The integration of descending and ascending input through these interneurons drives LTP in the spinal cord necessary for recovery. Identification of spinal interneurons that specifically mediate reaching and grasping behaviors will be critical targets for improving therapeutic outcomes.

In humans, anatomical plasticity is often inferred from motor and sensory evoked potential recordings of neural activity, electromyographic (EMG) recordings of motor output, or other neuroimaging techniques. Transmagnetic stimulation (TMS) of different muscle groups has been used to show changes (or the lack thereof) in cortical motor mapping or alterations in root sparing, and to estimate CST innervation of different spinal cord segments following injury [110,111]. Comparisons between stroke and SCI research are useful for understanding the role of upper motor neurons and CNS plasticity. For example, a recent study has shown adaptive strategies of motor unit recruitment from the contralesional RST following stroke that prioritize elbow, wrist, and finger flexion synergy over dextrous digit manipulation, which limits functional recovery [112]. Similarly, muscle groups with spared RST input following SCI have been shown to be stronger than those without [113].

### 3.2. Neuromodulation to Drive Descending Plasticity after SCI

Because of the importance of dexterity in independence, a major focus of clinical research has been specifically on hand function. Upper extremity function in people with cervical SCI varies greatly. The level and severity of injury determines the loss of function, which dictates the individual’s ability to participate in physical rehabilitation. The SCI individual’s engagement and the mode of rehabilitation influences the rate and degree of recovery [114,115]. Numerous physical therapy and rehabilitation paradigms exist, such as context-dependent reaching and grasping, object manipulation tasks, strength training and neurostimulation [116,117,118]. Kinematic analysis of the clinical population has been rigorously performed to categorize several key features of both compensatory and regained upper extremity movement, which shows that existing treatments are not wholly effective at restoring function [119]. Devices are under development to measure improvements in hand motor control remotely [120].

Neuromodulation of spared connections between the CST and RST caudal to an injury has been shown in rodents to precipitate reorganization and functional recovery [121]. A series of studies from the Buford lab [122,123,124] illustrated the reciprocal control of upper limb flexor and extensor muscles bilaterally. The related firing patterns of the pontomedullary reticular formation may play a role in compensatory neural control of upper extremity function after SCI. Additional connections between RST neurons and propriospinal interneurons within the spinal cord allow for descending input to circumnavigate lesion sites by crossing midline twice [125,126,127].

Reminiscent of forced-use paradigms popular for post-stroke rehabilitation [128,129], individuals suffering from lost hand dexterity will rely on compensatory movements, which potentially limit complete restoration of hand motor function [130]. In individuals with no remaining motor control of the forearm, brain computer interfaces are a promising alternative strategy for regaining independence. These devices are engineered to either compensate for lost circuitry or to drive anatomical plasticity in efforts to establish new connections or to strengthen existing but weak synaptic circuits. Brain computer interfaces exist that allow for reaching and grasping with robotic arms [131]. Current research is underway to augment these devices to have digit specific movement based on cortical activity [132], as well as to enhance plastic changes through neuromodulation using cortical stimulation, spinal stimulation, and tactile feedback [118,133,134,135,136].

### 3.3. Primary Afferent Plasticity

Primary afferent fibers supply information to spinal cord neurons about proprioception as well as information about object size, shape, and texture that are important for successful grasping of an object [61,137]. Axons from mechanosensitive and proprioceptive neurons ascend supraspinally in the ipsilateral dorsal columns and send collateral axons into motor centers of the spinal cord. Comprehensive reviews of primary afferent input to the spinal cord, as well as its targeting of spinal interneurons, can be found by Gatto et al. [138] as well as Abraira and Ginty [139]. These interneurons in the deep dorsal horn have been shown to regulate sensory input to motor circuits via presynaptic inhibition [138,140,141]. In humans, primary afferent input is integrated with motor commands via presynaptic inhibition in the cord from primary afferents and interneurons [142]. Thus, primary afferent input is one neural substrate that can modulate spinal interneurons involved in the reach-to-grasp movement.

The first example of anatomical plasticity of primary afferent fibers in response to injury was published by Liu and Chambers in 1958 [143]. In this seminal work, the authors demonstrated that dorsal root injury caused collateral sprouting of adjacent dorsal root axons into the dorsal horn of the cat. A series of studies by Murray and Goldberger [144,145,146] further demonstrated that collateral sprouting of primary afferent fibers resulted in recovery of motor function after either dorsal root or spinal cord injury. Others report sprouting of intact propriospinal interneurons following spinal hemisection as a neural mechanism of locomotor recovery [101,102,103]. The anatomical changes seen in these experiments were not a regenerative effort of damaged neurons, but rather a growth effort of undamaged and intact neurons that corresponded to improvements in function. Altered primary afferent input may be transmitted to motor neurons through deep dorsal horn interneurons, and membrane properties of these deep dorsal horn interneurons rostral and caudal to SCI demonstrated decreased input resistance and rheobase, indicating a hyperexcitable state [147].

Notably, the primary sensory neurons of the dorsal root ganglia (DRG) do not merely transmit feedback information to motor systems, but certain subclasses, called nociceptors, can transmit pain and temperature information from the peripheral tissues to the superficial or deep dorsal horn of the spinal cord. In addition, these nociceptive neurons extend axon collaterals to grey matter laminae in neighboring spinal cord segments via Lissauer’s tract. Detailed descriptions of dorsal horn neuron populations that receive nociceptive afferent input can be found in Peirs et al. [148]. Cutaneous nociceptive primary afferent fibers are analogous to Type III (Aδ) and Type IV (C) primary afferent fibers from the muscle, which innervate slightly deeper into the spinal cord [149]. Importantly, nociceptors can indirectly act on motoneurons at the spinal level. The withdrawal reflex is an example of a nociceptor mediated motor output. This polysynaptic reflex takes afferent input from predominantly Aδ fibers and passes it through a series of interneurons to cause ipsilateral flexion and contralateral extension of the limbs [34]. A recent study showed that C-fibers are responsible for a second, delayed phase of activity in the withdrawal reflex [150]. This suggests that C-fibers are also capable of reaching motoneurons via interneurons. Furthermore, the Type III and IV muscle afferents have been shown to modulate motor unit recruitment when firing during exercise-induced fatigue [151]. This firing has a unique modulatory effect because silent nociceptors become sensitive to stretch in the presence of exercise related metabolites [149]. This evidence shows that nociceptors not only are capable of influencing motor control at the spinal level, but also that different circumstances change how much they are involved.

After experimental SCI, nociceptive primary afferent fibers display a robust and maladaptive increase in their terminal arborizations in the dorsal horn [152,153] and display hyperexcitability and increased spontaneous activity [154]. Analysis of sensation in individuals with cervical SCI suggest that intrinsic changes in primary sensory neurons could, in part, mediate the return of functional sensation, as well as maladaptive allodynia and hyperalgesia, often observed over time following SCI [155]. Of course, these morphological and functional changes in nociceptive primary afferent input are associated with the development of neuropathic pain, but nociceptive information is also supplied for tissue and joint protection via reflex arcs to modulate normal motor circuit function and motor output. Therefore, aberrant plasticity of nociceptive afferents may be detrimental to functional recovery following SCI.

## 4. Rehabilitation, and Afferent Driven Plasticity for Reaching and Grasping

The current standard of care for tetraplegia after SCI includes a wide range of neuromodulation and physical therapy with varying success [116]. Intraspinal microstimulation and transcutaneous stimulation have been used to augment reaching and grasping after SCI and to have persistent effects, which indicate a promotion in neuroplasticity [117,156]. Other stimulation targets after injury focus on the periphery and exist to aid voluntary movement. These devices provide scaled strength to the hand when volitional movement is detected via tactile pressure sensors to facilitate use of the individual’s hand, which promotes both independence and rehabilitation-driven plasticity [157]. Similar devices activate an exoskeleton to aid in arm usage based on EMG recordings of volitional movement [158]. An alternative to task specific training is neurofeedback training using magnetoencephalography. Tetraplegic individuals use this technique to learn to control a virtual hand using the same brain waves they would use to move their own hand. This training led to improvements in grip strength [159]. Efforts to drive LTP and increase synaptic efficiency involve paired associative stimulation in which synchronized stimulation of the cortex with transcranial magnetic stimulation and peripheral nerve electrical stimulation have yielded positive outcomes in forelimb recovery [160,161]. Robotic assistance is also being combined with muscle stimulation to promote recovery and accuracy of the exoskeletal assisted movements [162]. Studies are underway to use multi-modal decoding algorithms to overcome the major hurdle of accurately determining the intended movement based on EMG output [163]. Combinatorial therapies yield the greatest results [164].

Rehabilitative strategies capitalize on the ability of the primary afferent to adapt and change in order to improve function after an injury. These physical therapies can include task specific training, such as repetitively reaching for, grasping, and manipulating objects. Additional therapies include resistance or aerobic training, as well as range-of-motion movements and stretching. Many of these are emulated in the laboratory. It is well established that task specific training improves reaching and grasping behavior after injury [165,166,167,168]. Cutaneous afferent input has been shown to significantly improve behavioral restoration and drive CNS plasticity following a lesion of the dorsal column [60]. SCI and subsequent treadmill training alter spinal interneuron excitability [147,169]. In addition, our lab and others have shown that early aerobic exercise can prevent pain development and reduce aberrant changes in nociceptor distribution and density within the superficial dorsal horn [170,171,172], suggesting a convergence of the nociceptive system and sensorimotor feedback systems within the dorsal horn to influence motor efferent output and behavior.

In 2018, Keller, et al. [173] investigated the EMG patterns in hindlimb muscles of SCI rats that underwent a daily range-of-motion stretching therapy. After several consecutive days of stretching, locomotor ability declined, and EMG recordings revealed increased clonus-like contractions during stretching [173]. This irregular muscle firing due to the stretching may explain the emergence of the irregular stepping patterns. A follow-up study demonstrated that ablation of nociceptors reduced stretching-induced locomotor deficits [174]. Nociceptors are often considered to be isolated in the sensory system, with the only overlap into motor systems occurring in withdrawal reflex circuitry. Even then, the withdrawal reflex is mediated via A fibers (type III fibers), while much of the work here is focused on C-fibers (likely type IV fibers). Collectively, this work shows that nociceptors might play a different and more influential role on motor output after SCI.

Numerous techniques are being used and tested for human rehabilitation after SCI, including a host of task specific and guided movement exercises. At the forefront, is a focus on accessibility and enhancing plasticity. Novel technologies, such as virtual reality (VR), allow individuals with SCI to perform more complex physical therapy independently and from home. VR devices are capable of delivering multiple physical therapy movement routines and accurately tracking associated hand kinematics [175]. Since VR technology only became available a few years ago, there is limited evidence demonstrating that VR therapy may be more beneficial than conventional physical therapy. However, the flexibility and affordability of VR devices and their accuracy in tracking movements will likely drive further research and development of this technology [176].

## 5. Conclusions

Neuroplasticity is a robust mechanism by which the central nervous system attempts to adapt to a structural or chemical disruption of functional connections between neurons. This adaptation is a prominent feature during neurodevelopment and is found in learning and memory. Following damage to nervous tissue, such as spinal cord injury, spontaneous plasticity may be initiated. Spontaneous plasticity can be maladaptive, as in the case of chronic neuropathic pain, but it can also lead to the recovery of lost function. Sprouting, synaptogenesis, synaptic plasticity, and pruning of connections between afferent fibers, interneurons, and motoneurons of ascending and descending tracts all play a role in mediating meaningful recovery after injury. Minor pathways often compensate for damaged major pathways, but interventions can lead to damaged axons regenerating across the lesion to improve function [14].

One of the major drives of modern neuroscience research is directed towards harnessing the power of neuroplasticity through rehabilitation. The human CNS has limited ability to spontaneously recover, however, many of the underlying mechanisms that allow recovery in other animals are still present. The rehabilitation research field attempts to use physical training to force central drive and primary afferent input into the spinal cord. Significantly, this shows that spontaneous plasticity within segmental spinal cord circuitry can be enhanced by rehabilitation. This gives hope for future research into the field, as rehabilitative techniques are optimized and even combined with pharmacological or neuromodulatory treatments. Not only do future studies need to continue to perfect treatments, but they also need to consider the fundamental changes in the nervous system, especially the spinal cord after injury. Certain cells, such as C-fiber nociceptors, are often neglected when considering movement or modeling motor circuitry. This has changed as alternatives to opioids for chronic pain treatment gain focus. Certainly, further research into the specific role of subclasses of primary afferent feedback after injury, especially nociceptors, is warranted.

## References

[1] Donnelly D.J., Popovich P.G. (2008). Inflammation and its role in neuroprotection, axonal regeneration and functional recovery after spinal cord injury. Exp. Neurol..

[2] Detloff M.R., Fisher L.C., McGaughy V., Longbrake E.E., Popovich P.G., Basso D.M. (2008). Remote activation of microglia and pro-inflammatory cytokines predict the onset and severity of below-level neuropathic pain after spinal cord injury in rats. Exp. Neurol..

[3] Gensel J.C., Zhang B. (2015). Macrophage activation and its role in repair and pathology after spinal cord injury. Brain Res..

[4] Kopper T.J., Gensel J.C. (2018). Myelin as an inflammatory mediator: Myelin interactions with complement, macrophages, and microglia in spinal cord injury. J. Neurosci. Res..

[5] Chhaya S.J., Quiros-Molina D., Tamashiro-Orrego A.D., Houle J.D., Detloff M.R. (2019). Exercise-Induced Changes to the Macrophage Response in the Dorsal Root Ganglia Prevent Neuropathic Pain after Spinal Cord Injury. J. Neurotraum..

[6] Kroner A., Almanza J.R. (2019). Role of microglia in spinal cord injury. Neurosci. Lett..

[7] O’Shea T.M., Burda J.E., Sofroniew M.V. (2017). Cell biology of spinal cord injury and repair. J. Clin. Investig..

[8] Tran A.P., Warren P.M., Silver J. (2021). New insights into glial scar formation after spinal cord injury. Cell Tissue Res..

[9] Silver J., Miller J.H. (2004). Regeneration beyond the glial scar. Nat. Rev. Neurosci..

[10] Reier P.J., Houle J.D. (1988). The glial scar: Its bearing on axonal elongation and transplantation approaches to CNS repair. Adv. Neurol..

[11] Snow D.M., Brown E.M., Letourneau P.C. (1996). Growth cone behavior in the presence of soluble chondroitin sulfate proteoglycan (CSPG), compared to behavior on CSPG bound to laminin or fibronectin. Int. J. Dev. Neurosci..

[12] Fawcett J.W., Asher R.A. (1999). The glial scar and central nervous system repair. Brain Res. Bull..

[13] Herrmann J.E., Imura T., Song B., Qi J., Ao Y., Nguyen T.K., Korsak R.A., Takeda K., Akira S., Sofroniew M.V. (2008). STAT3 is a critical regulator of astrogliosis and scar formation after spinal cord injury. J. Neurosci..

[14] Tom V.J., Houle J.D. (2008). Intraspinal microinjection of chondroitinase ABC following injury promotes axonal regeneration out of a peripheral nerve graft bridge. Exp. Neurol..

[15] Tran A.P., Warren P.M., Silver J. (2018). The Biology of Regeneration Failure and Success After Spinal Cord Injury. Physiol. Rev..

[16] O’Reilly M.L., Tom V.J. (2020). Neuroimmune System as a Driving Force for Plasticity Following CNS Injury. Front. Cell Neurosci..

[17] Anderson M.A., Burda J.E., Ren Y., Ao Y., O’Shea T.M., Kawaguchi R., Coppola G., Khakh B.S., Deming T.J., Sofroniew M.V. (2016). Astrocyte scar formation aids central nervous system axon regeneration. Nature.

[18] Lee J.K., Geoffroy C.G., Chan A.F., Tolentino K.E., Crawford M.J., Leal M.A., Kang B., Zheng B. (2010). Assessing spinal axon regeneration and sprouting in Nogo-, MAG-, and OMgp-deficient mice. Neuron.

[19] National Spinal Cord Injury Statistical Center (2021). Facts and Figures at a Glance.

[20] Xiao Z., Tang F., Zhao Y., Han G., Yin N., Li X., Chen B., Han S., Jiang X., Yun C. (2018). Significant Improvement of Acute Complete Spinal Cord Injury Patients Diagnosed by a Combined Criteria Implanted with NeuroRegen Scaffolds and Mesenchymal Stem Cells. Cell Transplant..

[21] Flesher S.N., Downey J.E., Weiss J.M., Hughes C.L., Herrera A.J., Tyler-Kabara E.C., Boninger M.L., Collinger J.L., Gaunt R.A. (2021). A brain-computer interface that evokes tactile sensations improves robotic arm control. Science.

[22] Goshgarian H.G. (1979). Developmental plasticity in the respiratory pathway of the adult rat. Exp. Neurol..

[23] Zimmer M.B., Goshgarian H.G. (2007). GABA, not glycine, mediates inhibition of latent respiratory motor pathways after spinal cord injury. Exp. Neurol..

[24] Lee K.Z., Dougherty B.J., Sandhu M.S., Lane M.A., Reier P.J., Fuller D.D. (2013). Phrenic motoneuron discharge patterns following chronic cervical spinal cord injury. Exp. Neurol..

[25] Hachem L.D., Ahuja C.S., Fehlings M.G. (2017). Assessment and management of acute spinal cord injury: From point of injury to rehabilitation. J. Spinal Cord Med..

[26] Wolpaw J.R., Lee C.L., Carp J.S. (1991). Operantly conditioned plasticity in spinal cord. Ann. N. Y. Acad. Sci..

[27] Gomes-Osman J., Field-Fote E.C. (2015). Cortical vs. afferent stimulation as an adjunct to functional task practice training: A randomized, comparative pilot study in people with cervical spinal cord injury. Clin. Rehabil..

[28] Field-Fote E.C. (2015). Exciting recovery: Augmenting practice with stimulation to optimize outcomes after spinal cord injury. Prog. Brain Res..

[29] Schildt C.J., Thomas S.H., Powell E.S., Sawaki L., Sunderam S. Closed-loop afferent electrical stimulation for recovery of hand function in individuals with motor incomplete spinal injury: Early clinical results. Proceedings of the 2016 38th Annual International Conference of the IEEE Engineering in Medicine and Biology Society (EMBC).

[30] Fee M.S. (2014). The role of efference copy in striatal learning. Curr. Opin. Neurobiol..

[31] Alstermark B., Ekerot C.F. (2015). The lateral reticular nucleus; integration of descending and ascending systems regulating voluntary forelimb movements. Front. Comput. Neurosci..

[32] Person A.L. (2019). Corollary Discharge Signals in the Cerebellum. Biol. Psychiatry Cogn. Neurosci. Neuroimaging.

[33] Van Kemenade B.M., Arikan B.E., Podranski K., Steinstrater O., Kircher T., Straube B. (2019). Distinct Roles for the Cerebellum, Angular Gyrus, and Middle Temporal Gyrus in Action-Feedback Monitoring. Cereb. Cortex.

[34] Kandel E., Koster J., Mack S., Siegelbaum S. (2013). Principles of Neural Science.

[35] Sweatt J.D. (2016). Neural plasticity and behavior—Sixty years of conceptual advances. J. Neurochem..

[36] Bliss T.V., Lomo T. (1973). Long-lasting potentiation of synaptic transmission in the dentate area of the anaesthetized rabbit following stimulation of the perforant path. J. Physiol..

[37] Hebb D.O. (1949). The Organization of Behavior: A Neuropsychological Theory.

[38] Zenke F., Gerstner W. (2017). Hebbian plasticity requires compensatory processes on multiple timescales. Philos. Trans. R. Soc. Lond. B Biol. Sci..

[39] Diering G.H., Huganir R.L. (2018). The AMPA Receptor Code of Synaptic Plasticity. Neuron.

[40] Dittman J.S., Ryan T.A. (2019). The control of release probability at nerve terminals. Nat. Rev. Neurosci..

[41] Harris K.M. (2020). Structural LTP: From synaptogenesis to regulated synapse enlargement and clustering. Curr. Opin. Neurobiol..

[42] Zucker R.S., Regehr W.G. (2002). Short-term synaptic plasticity. Annu. Rev. Physiol..

[43] Jackman S.L., Regehr W.G. (2017). The Mechanisms and Functions of Synaptic Facilitation. Neuron.

[44] Luscher C., Malenka R.C. (2012). NMDA receptor-dependent long-term potentiation and long-term depression (LTP/LTD). Cold Spring Harb. Perspect. Biol..

[45] Koike-Tani M., Kanda T., Saitoh N., Yamashita T., Takahashi T. (2008). Involvement of AMPA receptor desensitization in short-term synaptic depression at the calyx of Held in developing rats. J. Physiol..

[46] Herring B.E., Nicoll R.A. (2016). Long-Term Potentiation: From CaMKII to AMPA Receptor Trafficking. Annu. Rev. Physiol..

[47] Grasselli G., Hansel C. (2014). Cerebellar long-term potentiation: Cellular mechanisms and role in learning. Int. Rev. Neurobiol..

[48] Wolpaw J.R. (2007). Spinal cord plasticity in acquisition and maintenance of motor skills. Acta Physiol..

[49] Roth R.H., Cudmore R.H., Tan H.L., Hong I., Zhang Y., Huganir R.L. (2020). Cortical Synaptic AMPA Receptor Plasticity during Motor Learning. Neuron.

[50] Adkins D.L., Boychuk J., Remple M.S., Kleim J.A. (2006). Motor training induces experience-specific patterns of plasticity across motor cortex and spinal cord. J. Appl. Physiol..

[51] Hoy K.C., Huie J.R., Grau J.W. (2013). AMPA receptor mediated behavioral plasticity in the isolated rat spinal cord. Behav. Brain Res..

[52] Ganzer P.D., Beringer C.R., Shumsky J.S., Nwaobasi C., Moxon K.A. (2018). Serotonin receptor and dendritic plasticity in the spinal cord mediated by chronic serotonergic pharmacotherapy combined with exercise following complete SCI in the adult rat. Exp. Neurol..

[53] Lisman J. (2017). Glutamatergic synapses are structurally and biochemically complex because of multiple plasticity processes: Long-term potentiation, long-term depression, short-term potentiation and scaling. Philos. Trans. R. Soc. Lond. B Biol. Sci.

[54] Purkey A.M., Dell’Acqua M.L. (2020). Phosphorylation-Dependent Regulation of Ca(2+)-Permeable AMPA Receptors During Hippocampal Synaptic Plasticity. Front. Synaptic. Neurosci..

[55] Kulik Y.D., Watson D.J., Cao G., Kuwajima M., Harris K.M. (2019). Structural plasticity of dendritic secretory compartments during LTP-induced synaptogenesis. Elife.

[56] Whishaw I.Q., Pellis S.M. (1990). The structure of skilled forelimb reaching in the rat: A proximally driven movement with a single distal rotatory component. Behav. Brain Res..

[57] Whishaw I.Q., Pellis S.M., Gorny B., Kolb B., Tetzlaff W. (1993). Proximal and distal impairments in rat forelimb use in reaching follow unilateral pyramidal tract lesions. Behav. Brain Res..

[58] Haines D.E. (2000). Neuroanatomy: An Atlas of Structures, Sections, and Systems.

[59] Pizzimenti M.A., Darling W.G., Rotella D.L., McNeal D.W., Herrick J.L., Ge J., Stilwell-Morecraft K.S., Morecraft R.J. (2007). Measurement of reaching kinematics and prehensile dexterity in nonhuman primates. J. Neurophysiol..

[60] Qi H.X., Gharbawie O.A., Wynne K.W., Kaas J.H. (2013). Impairment and recovery of hand use after unilateral section of the dorsal columns of the spinal cord in squirrel monkeys. Behav. Brain Res..

[61] Geed S., McCurdy M.L., van Kan P.L. (2017). Neuronal Correlates of Functional Coupling between Reach- and Grasp-Related Components of Muscle Activity. Front. Neural. Circuits.

[62] Baker S.N. (2011). The primate reticulospinal tract, hand function and functional recovery. J. Physiol..

[63] Sathyamurthy A., Barik A., Dobrott C.I., Matson K.J.E., Stoica S., Pursley R., Chesler A.T., Levine A.J. (2020). Cerebellospinal Neurons Regulate Motor Performance and Motor Learning. Cell Rep..

[64] McKenna J.E., Prusky G.T., Whishaw I.Q. (2000). Cervical motoneuron topography reflects the proximodistal organization of muscles and movements of the rat forelimb: A retrograde carbocyanine dye analysis. J. Comp. Neurol..

[65] Klein A., Sacrey L.A., Dunnett S.B., Whishaw I.Q., Nikkhah G. (2011). Proximal movements compensate for distal forelimb movement impairments in a reach-to-eat task in Huntington’s disease: New insights into motor impairments in a real-world skill. Neurobiol. Dis..

[66] Klein A., Sacrey L.A., Whishaw I.Q., Dunnett S.B. (2012). The use of rodent skilled reaching as a translational model for investigating brain damage and disease. Neurosci. Biobehav. Rev..

[67] Krisa L., Runyen M., Detloff M.R. (2018). Translational Challenges of Rat Models of Upper Extremity Dysfunction After Spinal Cord Injury. Top. Spinal Cord Inj. Rehabil..

[68] Gallegos C., Carey M., Zheng Y., He X., Cao Q.L. (2020). Reaching and Grasping Training Improves Functional Recovery After Chronic Cervical Spinal Cord Injury. Front. Cell Neurosci..

[69] Montoya C.P., Campbell-Hope L.J., Pemberton K.D., Dunnett S.B. (1991). The “staircase test”: A measure of independent forelimb reaching and grasping abilities in rats. J. Neurosci. Methods.

[70] Sindhurakar A., Butensky S.D., Meyers E., Santos J., Bethea T., Khalili A., Sloan A.P., Rennaker R.L., Carmel J.B. (2017). An Automated Test of Rat Forelimb Supination Quantifies Motor Function Loss and Recovery After Corticospinal Injury. Neurorehabil. Neural. Repair..

[71] Irvine K.A., Ferguson A.R., Mitchell K.D., Beattie S.B., Beattie M.S., Bresnahan J.C. (2010). A novel method for assessing proximal and distal forelimb function in the rat: The Irvine, Beatties and Bresnahan (IBB) forelimb scale. J. Vis. Exp..

[72] Irvine K.A., Ferguson A.R., Mitchell K.D., Beattie S.B., Lin A., Stuck E.D., Huie J.R., Nielson J.L., Talbott J.F., Inoue T. (2014). The Irvine, Beatties, and Bresnahan (IBB) Forelimb Recovery Scale: An Assessment of Reliability and Validity. Front. Neurol..

[73] Ballermann M., Metz G.A., McKenna J.E., Klassen F., Whishaw I.Q. (2001). The pasta matrix reaching task: A simple test for measuring skilled reaching distance, direction, and dexterity in rats. J. Neurosci. Methods.

[74] Bertelli J.A., Mira J.C. (1993). Behavioral evaluating methods in the objective clinical assessment of motor function after experimental brachial plexus reconstruction in the rat. J. Neurosci. Methods.

[75] Anderson K.D., Gunawan A., Steward O. (2005). Quantitative assessment of forelimb motor function after cervical spinal cord injury in rats: Relationship to the corticospinal tract. Exp. Neurol..

[76] Khaing Z.Z., Geissler S.A., Jiang S., Milman B.D., Aguilar S.V., Schmidt C.E., Schallert T. (2012). Assessing forelimb function after unilateral cervical spinal cord injury: Novel forelimb tasks predict lesion severity and recovery. J. Neurotraum..

[77] McCann M.M., Fisher K.M., Ahloy-Dallaire J., Darian-Smith C. (2020). Somatosensory corticospinal tract axons sprout within the cervical cord following a dorsal root/dorsal column spinal injury in the rat. J. Comp. Neurol..

[78] Ortiz-Rosario A., Berrios-Torres I., Adeli H., Buford J.A. (2014). Combined corticospinal and reticulospinal effects on upper limb muscles. Neurosci. Lett..

[79] Baker S.N., Zaaimi B., Fisher K.M., Edgley S.A., Soteropoulos D.S. (2015). Pathways mediating functional recovery. Prog. Brain Res..

[80] Palmer E., Ashby P. (1992). Corticospinal projections to upper limb motoneurones in humans. J. Physiol..

[81] De Noordhout A.M., Rapisarda G., Bogacz D., Gerard P., De Pasqua V., Pennisi G., Delwaide P.J. (1999). Corticomotoneuronal synaptic connections in normal man: An electrophysiological study. Brain.

[82] Tazoe T., Perez M.A. (2017). Cortical and reticular contributions to human precision and power grip. J. Physiol..

[83] Schrimsher G.W., Reier P.J. (1993). Forelimb motor performance following dorsal column, dorsolateral funiculi, or ventrolateral funiculi lesions of the cervical spinal cord in the rat. Exp. Neurol..

[84] Morecraft R.J., Ge J., Stilwell-Morecraft K.S., McNeal D.W., Pizzimenti M.A., Darling W.G. (2013). Terminal distribution of the corticospinal projection from the hand/arm region of the primary motor cortex to the cervical enlargement in rhesus monkey. J. Comp. Neurol..

[85] Oudega M., Perez M.A. (2012). Corticospinal reorganization after spinal cord injury. J. Physiol..

[86] Weidner N., Ner A., Salimi N., Tuszynski M.H. (2001). Spontaneous corticospinal axonal plasticity and functional recovery after adult central nervous system injury. Proc. Natl. Acad. Sci. USA.

[87] Nakagawa H., Ninomiya T., Yamashita T., Takada M. (2015). Reorganization of corticospinal tract fibers after spinal cord injury in adult macaques. Sci. Rep..

[88] Armstrong D.M. (1986). Supraspinal contributions to the initiation and control of locomotion in the cat. Prog. Neurobiol..

[89] Shik M.L., Orlovsky G.N. (1976). Neurophysiology of locomotor automatism. Physiol. Rev..

[90] Dougherty K.J., Kiehn O. (2010). Functional organization of V2a-related locomotor circuits in the rodent spinal cord. Ann. N. Y. Acad. Sci..

[91] Alaynick W.A., Jessell T.M., Pfaff S.L. (2011). SnapShot: Spinal cord development. Cell.

[92] Lu D.C., Niu T., Alaynick W.A. (2015). Molecular and cellular development of spinal cord locomotor circuitry. Front. Mol. Neurosci..

[93] Rybak I.A., Dougherty K.J., Shevtsova N.A. (2015). Organization of the Mammalian Locomotor CPG: Review of Computational Model and Circuit Architectures Based on Genetically Identified Spinal Interneurons(1,2,3). eNeuro.

[94] Flynn J.R., Conn V.L., Boyle K.A., Hughes D.I., Watanabe M., Velasquez T., Goulding M.D., Callister R.J., Graham B.A. (2017). Anatomical and Molecular Properties of Long Descending Propriospinal Neurons in Mice. Front. Neuroanat..

[95] Zholudeva L.V., Qiang L., Marchenko V., Dougherty K.J., Sakiyama-Elbert S.E., Lane M.A. (2018). The Neuroplastic and Therapeutic Potential of Spinal Interneurons in the Injured Spinal Cord. Trends Neurosci..

[96] Dobrott C.I., Sathyamurthy A., Levine A.J. (2019). Decoding Cell Type Diversity Within the Spinal Cord. Curr. Opin. Physiol..

[97] Zholudeva L.V., Abraira V.E., Satkunendrarajah K., McDevitt T.C., Goulding M.D., Magnuson D.S.K., Lane M.A. (2021). Spinal Interneurons as Gatekeepers to Neuroplasticity after Injury or Disease. J. Neurosci..

[98] Abraira V.E., Kuehn E.D., Chirila A.M., Springel M.W., Toliver A.A., Zimmerman A.L., Orefice L.L., Boyle K.A., Bai L., Song B.J. (2017). The Cellular and Synaptic Architecture of the Mechanosensory Dorsal Horn. Cell.

[99] Gatto G., Bourane S., Ren X., Di Costanzo S., Fenton P.K., Halder P., Seal R.P., Goulding M.D. (2021). A Functional Topographic Map for Spinal Sensorimotor Reflexes. Neuron.

[100] Pocratsky A.M., Shepard C.T., Morehouse J.R., Burke D.A., Riegler A.S., Hardin J.T., Beare J.E., Hainline C., States G.J., Brown B.L. (2020). Long ascending propriospinal neurons provide flexible, context-specific control of interlimb coordination. Elife.

[101] Bareyre F.M., Kerschensteiner M., Raineteau O., Mettenleiter T.C., Weinmann O., Schwab M.E. (2004). The injured spinal cord spontaneously forms a new intraspinal circuit in adult rats. Nat. Neurosci..

[102] Raineteau O., Schwab M.E. (2001). Plasticity of motor systems after incomplete spinal cord injury. Nat. Rev. Neurosci..

[103] Courtine G., Song B., Roy R.R., Zhong H., Herrmann J.E., Ao Y., Qi J., Edgerton V.R., Sofroniew M.V. (2008). Recovery of supraspinal control of stepping via indirect propriospinal relay connections after spinal cord injury. Nat. Med..

[104] Benthall K.N., Hough R.A., McClellan A.D. (2017). Descending propriospinal neurons mediate restoration of locomotor function following spinal cord injury. J. Neurophysiol..

[105] Morris R., Tosolini A.P., Goldstein J.D., Whishaw I.Q. (2011). Impaired arpeggio movement in skilled reaching by rubrospinal tract lesions in the rat: A behavioral/anatomical fractionation. J. Neurotraum..

[106] Hayashi M., Hinckley C.A., Driscoll S.P., Moore N.J., Levine A.J., Hilde K.L., Sharma K., Pfaff S.L. (2018). Graded Arrays of Spinal and Supraspinal V2a Interneuron Subtypes Underlie Forelimb and Hindlimb Motor Control. Neuron.

[107] Whishaw I.Q., Gorny B. (1994). Arpeggio and fractionated digit movements used in prehension by rats. Behav. Brain Res..

[108] Isa T., Kinoshita M., Nishimura Y. (2013). Role of Direct vs. Indirect Pathways from the Motor Cortex to Spinal Motoneurons in the Control of Hand Dexterity. Front. Neurol..

[109] Azim E., Jiang J., Alstermark B., Jessell T.M. (2014). Skilled reaching relies on a V2a propriospinal internal copy circuit. Nature.

[110] Brouwer B., Hopkins-Rosseel D.H. (1997). Motor cortical mapping of proximal upper extremity muscles following spinal cord injury. Spinal Cord.

[111] Calancie B., Alexeeva N., Broton J.G., Suys S., Hall A., Klose K.J. (1999). Distribution and latency of muscle responses to transcranial magnetic stimulation of motor cortex after spinal cord injury in humans. J. Neurotraum..

[112] McPherson J.G., Chen A., Ellis M.D., Yao J., Heckman C.J., Dewald J.P.A. (2018). Progressive recruitment of contralesional cortico-reticulospinal pathways drives motor impairment post stroke. J. Physiol..

[113] Sangari S., Perez M.A. (2020). Distinct Corticospinal and Reticulospinal Contributions to Voluntary Control of Elbow Flexor and Extensor Muscles in Humans with Tetraplegia. J. Neurosci..

[114] Ditunno J.F., Stover S.L., Freed M.M., Ahn J.H. (1992). Motor recovery of the upper extremities in traumatic quadriplegia: A multicenter study. Arch. Phys. Med. Rehabil..

[115] Ditunno J.F., Cohen M.E., Hauck W.W., Jackson A.B., Sipski M.L. (2000). Recovery of upper-extremity strength in complete and incomplete tetraplegia: A multicenter study. Arch. Phys. Med. Rehabil..

[116] Mateo S., Di Marco J., Cucherat M., Gueyffier F., Rode G. (2020). Inconclusive efficacy of intervention on upper-limb function after tetraplegia: A systematic review and meta-analysis. Ann. Phys. Rehabil. Med..

[117] Kasten M.R., Sunshine M.D., Secrist E.S., Horner P.J., Moritz C.T. (2013). Therapeutic intraspinal microstimulation improves forelimb function after cervical contusion injury. J. Neural. Eng..

[118] Ajiboye A.B., Willett F.R., Young D.R., Memberg W.D., Murphy B.A., Miller J.P., Walter B.L., Sweet J.A., Hoyen H.A., Keith M.W. (2017). Restoration of reaching and grasping movements through brain-controlled muscle stimulation in a person with tetraplegia: A proof-of-concept demonstration. Lancet.

[119] Mateo S., Roby-Brami A., Reilly K.T., Rossetti Y., Collet C., Rode G. (2015). Upper limb kinematics after cervical spinal cord injury: A review. J. Neuroeng. Rehabil..

[120] Tsai M.F., Wang R.H., Zariffa J. (2020). Generalizability of Hand-Object Interaction Detection in Egocentric Video across Populations with Hand Impairment. Annu. Int. Conf. IEEE Eng. Med. Biol. Soc..

[121] Asboth L., Friedli L., Beauparlant J., Martinez-Gonzalez C., Anil S., Rey E., Baud L., Pidpruzhnykova G., Anderson M.A., Shkorbatova P. (2018). Cortico-reticulo-spinal circuit reorganization enables functional recovery after severe spinal cord contusion. Nat. Neurosci..

[122] Davidson A.G., Buford J.A. (2006). Bilateral actions of the reticulospinal tract on arm and shoulder muscles in the monkey: Stimulus triggered averaging. Exp. Brain Res..

[123] Davidson A.G., Schieber M.H., Buford J.A. (2007). Bilateral spike-triggered average effects in arm and shoulder muscles from the monkey pontomedullary reticular formation. J. Neurosci..

[124] Herbert W.J., Davidson A.G., Buford J.A. (2010). Measuring the motor output of the pontomedullary reticular formation in the monkey: Do stimulus-triggered averaging and stimulus trains produce comparable results in the upper limbs?. Exp. Brain Res..

[125] Filli L., Engmann A.K., Zorner B., Weinmann O., Moraitis T., Gullo M., Kasper H., Schneider R., Schwab M.E. (2014). Bridging the gap: A reticulo-propriospinal detour bypassing an incomplete spinal cord injury. J. Neurosci..

[126] Zorner B., Bachmann L.C., Filli L., Kapitza S., Gullo M., Bolliger M., Starkey M.L., Rothlisberger M., Gonzenbach R.R., Schwab M.E. (2014). Chasing central nervous system plasticity: The brainstem’s contribution to locomotor recovery in rats with spinal cord injury. Brain.

[127] May Z., Fenrich K.K., Dahlby J., Batty N.J., Torres-Espin A., Fouad K. (2017). Following Spinal Cord Injury Transected Reticulospinal Tract Axons Develop New Collateral Inputs to Spinal Interneurons in Parallel with Locomotor Recovery. Neural. Plast.

[128] Taub E., Wolf S.L. (1997). Constraint Induced Movement Techniques To Facilitate Upper Extremity Use in Stroke Patients. Top. Stroke Rehabil..

[129] Dos Anjos S., Morris D., Taub E. (2020). Constraint-Induced Movement Therapy for Lower Extremity Function: Describing the LE-CIMT Protocol. Phys. Ther..

[130] Schneider S., Popp W.L., Brogioli M., Albisser U., Ortmann S., Velstra I.M., Demko L., Gassert R., Curt A. (2019). Predicting upper limb compensation during prehension tasks in tetraplegic spinal cord injured patients using a single wearable sensor. IEEE Int. Conf. Rehabil. Robot..

[131] Bockbrader M.A., Francisco G., Lee R., Olson J., Solinsky R., Boninger M.L. (2018). Brain Computer Interfaces in Rehabilitation Medicine. PM R.

[132] Jorge A., Royston D.A., Tyler-Kabara E.C., Boninger M.L., Collinger J.L. (2020). Classification of Individual Finger Movements Using Intracortical Recordings in Human Motor Cortex. Neurosurgery.

[133] Zimmermann J.B., Jackson A. (2014). Closed-loop control of spinal cord stimulation to restore hand function after paralysis. Front. Neurosci..

[134] Benavides F.D., Jo H.J., Lundell H., Edgerton V.R., Gerasimenko Y., Perez M.A. (2020). Cortical and Subcortical Effects of Transcutaneous Spinal Cord Stimulation in Humans with Tetraplegia. J. Neurosci..

[135] Anderson K.D., Bryden A.M., Moynahan M. (2019). Risk-benefit value of upper extremity function by an implanted electrical stimulation device targeting chronic cervical spinal cord injury. Spinal. Cord. Ser. Cases.

[136] Ganzer P.D., Colachis S.C.t., Schwemmer M.A., Friedenberg D.A., Dunlap C.F., Swiftney C.E., Jacobowitz A.F., Weber D.J., Bockbrader M.A., Sharma G. (2020). Restoring the Sense of Touch Using a Sensorimotor Demultiplexing Neural Interface. Cell.

[137] Geed S., van Kan P.L.E. (2017). Grasp-Based Functional Coupling Between Reach- and Grasp-Related Components of Forelimb Muscle Activity. J. Mot. Behav..

[138] Gatto G., Smith K.M., Ross S.E., Goulding M. (2019). Neuronal diversity in the somatosensory system: Bridging the gap between cell type and function. Curr. Opin. Neurobiol..

[139] Abraira V.E., Ginty D.D. (2013). The sensory neurons of touch. Neuron.

[140] Fink A.J., Croce K.R., Huang Z.J., Abbott L.F., Jessell T.M., Azim E. (2014). Presynaptic inhibition of spinal sensory feedback ensures smooth movement. Nature.

[141] Koch S.C., Del Barrio M.G., Dalet A., Gatto G., Gunther T., Zhang J., Seidler B., Saur D., Schule R., Goulding M. (2017). RORbeta Spinal Interneurons Gate Sensory Transmission during Locomotion to Secure a Fluid Walking Gait. Neuron.

[142] Knikou M., Chaudhuri D., Kay E., Schmit B.D. (2006). Pre- and post-alpha motoneuronal control of the soleus H-reflex during sinusoidal hip movements in human spinal cord injury. Brain Res..

[143] Liu C.N., Chambers W.W. (1958). Intraspinal sprouting of dorsal root axons; development of new collaterals and preterminals following partial denervation of the spinal cord in the cat. AMA Arch. Neurol. Psychiatry.

[144] Goldberger M.E., Murray M. (1974). Restitution of function and collateral sprouting in the cat spinal cord: The deafferented animal. J. Comp. Neurol..

[145] Murray M., Goldberger M.E. (1974). Restitution of function and collateral sprouting in the cat spinal cord: The partially hemisected animal. J. Comp. Neurol..

[146] Polistina D.C., Murray M., Goldberger M.E. (1990). Plasticity of dorsal root and descending serotoninergic projections after partial deafferentation of the adult rat spinal cord. J. Comp. Neurol..

[147] Rank M.M., Galea M.P., Callister R., Callister R.J. (2018). Is more always better? How different ’doses’ of exercise after incomplete spinal cord injury affects the membrane properties of deep dorsal horn interneurons. Exp. Neurol..

[148] Peirs C., Dallel R., Todd A.J. (2020). Recent advances in our understanding of the organization of dorsal horn neuron populations and their contribution to cutaneous mechanical allodynia. J. Neural. Transm..

[149] Laurin J., Pertici V., Dousset E., Marqueste T., Decherchi P. (2015). Group III and IV muscle afferents: Role on central motor drive and clinical implications. Neuroscience.

[150] Kimura S., Honda M., Tanabe M., Ono H. (2004). Noxious stimuli evoke a biphasic flexor reflex composed of A delta-fiber-mediated short-latency and C-fiber-mediated long-latency withdrawal movements in mice. J. Pharmacol. Sci..

[151] Zajac A., Chalimoniuk M., Maszczyk A., Golas A., Lngfort J. (2015). Central and Peripheral Fatigue During Resistance Exercise—A Critical Review. J. Hum. Kinet.

[152] Ondarza A.B., Ye Z., Hulsebosch C.E. (2003). Direct evidence of primary afferent sprouting in distant segments following spinal cord injury in the rat: Colocalization of GAP-43 and CGRP. Exp. Neurol..

[153] Detloff M.R., Quiros-Molina D., Javia A.S., Daggubati L., Nehlsen A.D., Naqvi A., Ninan V., Vannix K.N., McMullen M.K., Amin S. (2016). Delayed Exercise Is Ineffective at Reversing Aberrant Nociceptive Afferent Plasticity or Neuropathic Pain After Spinal Cord Injury in Rats. Neurorehabil. Neural. Repair..

[154] Bedi S.S., Yang Q., Crook R.J., Du J., Wu Z., Fishman H.M., Grill R.J., Carlton S.M., Walters E.T. (2010). Chronic spontaneous activity generated in the somata of primary nociceptors is associated with pain-related behavior after spinal cord injury. J. Neurosci..

[155] Kramer J.K., Taylor P., Steeves J.D., Curt A. (2010). Dermatomal somatosensory evoked potentials and electrical perception thresholds during recovery from cervical spinal cord injury. Neurorehabil. Neural. Repair.

[156] Inanici F., Samejima S., Gad P., Edgerton V.R., Hofstetter C.P., Moritz C.T. (2018). Transcutaneous Electrical Spinal Stimulation Promotes Long-Term Recovery of Upper Extremity Function in Chronic Tetraplegia. IEEE Trans. Neural. Syst. Rehabil. Eng..

[157] Osuagwu B.A.C., Timms S., Peachment R., Dowie S., Thrussell H., Cross S., Shirley R., Segura-Fragoso A., Taylor J. (2020). Home-based rehabilitation using a soft robotic hand glove device leads to improvement in hand function in people with chronic spinal cord injury:a pilot study. J. Neuroeng. Rehabil..

[158] McDonald C.G., Sullivan J.L., Dennis T.A., O’Malley M.K. (2020). A Myoelectric Control Interface for Upper-Limb Robotic Rehabilitation Following Spinal Cord Injury. IEEE Trans. Neural. Syst. Rehabil. Eng..

[159] Foldes S.T., Boninger M.L., Weber D.J., Collinger J.L. (2020). Effects of MEG-based neurofeedback for hand rehabilitation after tetraplegia: Preliminary findings in cortical modulations and grip strength. J. Neural. Eng..

[160] Roy F.D., Yang J.F., Gorassini M.A. (2010). Afferent regulation of leg motor cortex excitability after incomplete spinal cord injury. J. Neurophysiol..

[161] Rodionov A., Savolainen S., Kirveskari E., Makela J.P., Shulga A. (2019). Restoration of hand function with long-term paired associative stimulation after chronic incomplete tetraplegia: A case study. Spinal. Cord Ser. Cases.

[162] Dunkelberger N., Schearer E.M., O’Malley M.K. (2020). A review of methods for achieving upper limb movement following spinal cord injury through hybrid muscle stimulation and robotic assistance. Exp. Neurol..

[163] Corbett E.A., Sachs N.A., Kording K.P., Perreault E.J. (2014). Multimodal decoding and congruent sensory information enhance reaching performance in subjects with cervical spinal cord injury. Front. Neurosci..

[164] Lu X., Battistuzzo C.R., Zoghi M., Galea M.P. (2015). Effects of training on upper limb function after cervical spinal cord injury: A systematic review. Clin. Rehabil..

[165] Hurd C., Weishaupt N., Fouad K. (2013). Anatomical correlates of recovery in single pellet reaching in spinal cord injured rats. Exp. Neurol..

[166] Garcia-Alias G., Truong K., Shah P.K., Roy R.R., Edgerton V.R. (2015). Plasticity of subcortical pathways promote recovery of skilled hand function in rats after corticospinal and rubrospinal tract injuries. Exp. Neurol..

[167] Fenrich K.K., May Z., Torres-Espin A., Forero J., Bennett D.J., Fouad K. (2016). Single pellet grasping following cervical spinal cord injury in adult rat using an automated full-time training robot. Behav. Brain Res..

[168] Fenrich K.K., Hallworth B.W., Vavrek R., Raposo P.J.F., Misiaszek J.E., Bennett D.J., Fouad K., Torres-Espin A. (2021). Self-directed rehabilitation training intensity thresholds for efficient recovery of skilled forelimb function in rats with cervical spinal cord injury. Exp. Neurol..

[169] Garcia-Ramirez D.L., Ha N.T.B., Bibu S., Stachowski N.J., Dougherty K.J. (2021). Spinal cord injury alters spinal Shox2 interneurons by enhancing excitatory synaptic input and serotonergic modulation while maintaining intrinsic properties in mouse. J. Neurosci..

[170] Detloff M.R., Smith E.J., Quiros Molina D., Ganzer P.D., Houle J.D. (2014). Acute exercise prevents the development of neuropathic pain and the sprouting of non-peptidergic (GDNF- and artemin-responsive) c-fibers after spinal cord injury. Exp. Neurol..

[171] Nees T.A., Tappe-Theodor A., Sliwinski C., Motsch M., Rupp R., Kuner R., Weidner N., Blesch A. (2016). Early-onset treadmill training reduces mechanical allodynia and modulates calcitonin gene-related peptide fiber density in lamina III/IV in a mouse model of spinal cord contusion injury. Pain.

[172] Sliwinski C., Nees T.A., Puttagunta R., Weidner N., Blesch A. (2018). Sensorimotor Activity Partially Ameliorates Pain and Reduces Nociceptive Fiber Density in the Chronically Injured Spinal Cord. J. Neurotraum..

[173] Keller A.V., Rees K.M., Seibt E.J., Wood B.D., Wade A.D., Morehouse J., Shum-Siu A., Magnuson D.S.K. (2018). Electromyographic patterns of the rat hindlimb in response to muscle stretch after spinal cord injury. Spinal. Cord.

[174] Keller A.V., Hainline C., Rees K., Krupp S., Prince D., Wood B.D., Shum-Siu A., Burke D.A., Petruska J.C., Magnuson D.S.K. (2019). Nociceptor-dependent locomotor dysfunction after clinically-modeled hindlimb muscle stretching in adult rats with spinal cord injury. Exp. Neurol..

[175] De Los Reyes-Guzman A., Lozano-Berrio V., Alvarez-Rodriguez M., Lopez-Dolado E., Ceruelo-Abajo S., Talavera-Diaz F., Gil-Agudo A. (2021). RehabHand: Oriented-tasks serious games for upper limb rehabilitation by using Leap Motion Controller and target population in spinal cord injury. NeuroRehabilitation.

[176] De Miguel-Rubio A., Rubio M.D., Alba-Rueda A., Salazar A., Moral-Munoz J.A., Lucena-Anton D. (2020). Virtual Reality Systems for Upper Limb Motor Function Recovery in Patients With Spinal Cord Injury: Systematic Review and Meta-Analysis. JMIR Mhealth Uhealth.

